# Programmed repair of disease-causing UGA premature termination codons in mammalian brain

**DOI:** 10.1093/nar/gkag695

**Published:** 2026-07-16

**Authors:** Ahmad Al Saneh, Lionel Gissot, Christopher A Ahern

**Affiliations:** Department of Molecular Physiology and Biophysics, University of Iowa, Iowa City, IA 52242, United States; Department of Molecular Physiology and Biophysics, University of Iowa, Iowa City, IA 52242, United States; Department of Molecular Physiology and Biophysics, University of Iowa, Iowa City, IA 52242, United States

## Abstract

Protein-truncating variants caused by stop codons are the most prevalent class of rare variant mutations in neurodevelopmental diseases, with UGA codons being most common. Suppressor transfer RNA (sup-tRNA) has therapeutic potential for premature termination codon (PTC) rescue but has thus far underperformed by traditional AAV delivery platforms, and progress has been hampered by the lack of methods to non-invasively assess *in vivo* activity in mammalian brain. To fill this material gap, we utilize transcranial *in vivo* bioluminescence imaging data from a luciferase-UGA mouse model to optimize viral payloads with sup-tRNA genes. These data demonstrate that U6 promoter-driven and single-stranded AAV2/9 constructs show variable and dose-dependent activity, whereas self-complementary AAV2/9 with the tRNA in a minimal 100-bp genomic context provides broad and efficacious PTC rescue. Further, payload tRNA multiplexing and use of tRNA introns enable efficacy of low viral titers and sustained rescue. tRNA sequencing of scAAV-delivered Arg^UCA^ sup-tRNA in brain demonstrates no effects on endogenous tRNA levels, their acylation, or processing, and these features are also maintained in the delivered Arg^UCA^ sup-tRNA. Collectively, this work defines a scalable strategy for precision UGA PTC stop codon suppression, supporting development of durable genetic rescue therapies for neurodevelopmental disorders in the mammalian brain.

## Graphical Abstract

**Graphical Abstract:**
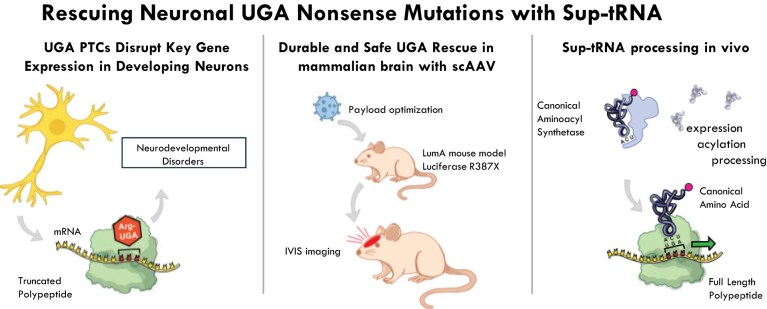

## Introduction

**Figure 1.**
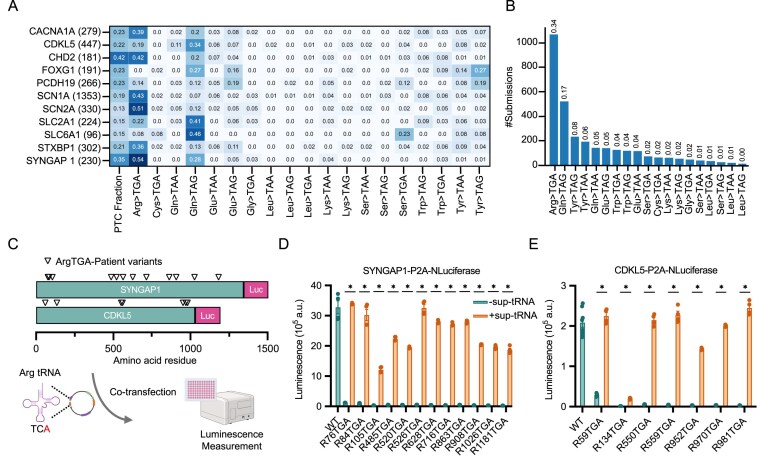
PTC variants of neurological disease and positionally agnostic rescue of UGA PTC stop codons in SYNGAP1 and CDKL5. (**A**) PTC landscape for a variety of neurological and neurodevelopmental disorders. Prevalence varies from 13% (*SCN2A*) to 42% (*CHD2*), with a majority caused by Arg CGA-to-TGA. (**B**) ClinVar submissions by stop codon type further demonstrate Arg^UGA^ as the dominant PTC. (**C**) Experimental layout to measure agnostic Arg^UGA^ PTC rescue in *SYNGAP1* and *CDKL5* across 19 variants from two genes: *SYNGAP1* (R76, R84, R105, R485, R520, R526, R628, R716, R863, R908, R1026, and R1181) and *CDKL5* (R59, R134, R550, R559, R952, R970, and R981). (**D, E**) Positional agnostic rescue in *SYNGAP1* and *CDKL5*. Only one site, R134TGA in CDKL5, demonstrated poor rescue, possibly owing to the position of this residue within a particularly complex protein fold within the CDKL5 kinase domain. Data were analyzed using multiple unpaired *t*-tests with Holm-Sidak correction (*P* < .00001, *n* = 4 per construct). Created in BioRender. Al saneh, A. (2026) https://BioRender.com/2ce96kj.

*De novo* protein truncations are common in neurodevelopmental disorders [1] and cause a range of genetic diseases, including intellectual disability [2], developmental delay [3, 4], autism [5, 6], epilepsy [7, 8], as well as sleep and autonomic dysfunction [5, 9]. One type of truncating variant is caused by the creation of a premature termination codon (PTC), accounting for 10%–15% of all inherited diseases [10]. Such PTCs are nearly absent in population studies, indicating that they are closely associated with disease [1]. While each PTC mutation is rare within any given disorder, a PTC can occur in principle throughout the entire coding sequence of a gene, thus posing a major therapeutic challenge. For this reason, efficient therapeutic strategies capable of rescuing a given PTC in a sequence-agnostic manner are needed. Small-molecule read-through therapies have performed poorly in clinical trials [11], possibly due to their propensity to incorporate near-cognate amino acids at the PTC [12, 13] and high off-target read-through of native stop codons [12–14]. Additionally, while gene editing can correct a specific PTC, individual validation of guide RNAs is necessary—a resource-intense effort that is currently not feasible for a multitude of rare (“N-of-1”) PTC variants. There is therefore a critical need for a broadly applicable treatment strategy that can correct PTCs, independent of the sequence context, with minimal toxicity in the brain. Notably, while 10 amino acids (and 19 codons) are 1 nt change away from becoming a PTC, the Arg CGA codon being mutated to the TGA stop codon represents the lion’s share of all PTCs (Fig 1A). This is due to a biochemical vulnerability in the CGA codon, where spontaneous deamination of cytosine to thymidine makes the CGA-to-TGA alternation the most common *de novo* PTC [15]. Therefore, a single approach with the ability to rescue individual Arg-TGA PTC codons [UGA in messenger RNA (mRNA)] has broad clinical potential.

### Suppressor tRNAs have the potential to rescue PTCs in a positionally agnostic manner

Suppressor transfer RNA (sup-tRNA) therapeutics have unique promise to promote protein restoration working at the level of translation of expressed mRNA [13, 16, 17]. We previously screened over 500 lab-engineered human sup-tRNAs to identify new variants that rescue PTCs and encode the correct amino acid [18, 19]. These engineered tRNAs have been used for the rescue of PTCs in human hERG potassium channels [19], the cystic fibrosis transmembrane conductance regulator protein [20], CDKL5 [21], and *in vivo* for Hurler syndrome caused by PTC variants [22, 23]. This strategy is disease-modifying because it restores full-length protein from PTC-containing mRNAs that would otherwise undergo premature translational termination. Each sup-tRNA has a substitution in the anticodon triplet which allows reading of a stop codon, while still being able to deliver the correct amino acid [18]. During translation, a sup-tRNA is accommodated by the ribosome at the PTC while delivering the correct amino acid to allow the ribosome to continue protein synthesis at that site and to proceed forward to codons past the PTC, generating a correctly translated and full-length protein. We and others have shown that this read-through of PTC does not obviously trigger read-through of native stop codons [18, 24] or ER stress [19, 25], although more data are needed to parse the mechanistic basis for this phenomena. Recent efforts with virally delivered anticodon-edited arginine tRNA have focused on UGA stop codons that cause Hurler syndrome (mucopolysaccharidosis type I, MPS I); however, rescue in these cases remains subtherapeutic [<8% of wild-type (WT) protein] potentially reflecting limitations in AAV titers and/or payload designs. Self-complementary AAV (scAAV) vectors have shown some promise with recent rescue of retinal PTC variants [25], but no such example has been broadly described for neurological disorders. Lastly, there has been widespread concern regarding any tRNA-based therapy that could unintentionally and generally interfere with native tRNA processing and acylation, leading to potentially broad off-target consequences.

We have previously identified the anticodon-modified Arg-TCT-1–1 tRNA gene as one of the strongest amongst the 28 human arginine tRNAs [18, 26]. Specifically, the single anticodon mutation UCU-to-UCA reveals potent UGA PTC suppression activity. We therefore first used tRNA-Arg-TCA-1–1(hereafter, Arg^UCA^ sup-tRNA) to demonstrate positionally agnostic rescue of patient UGA PTC variants in two genes, *SYNGAP1* and *CDKL5*, while analyzing the sequence context of each PTC. We then adopt a transgenic mouse model carrying a *Rosa26* firefly luciferase gene harboring an Arg-to-UGA PTC (p. R387X) to optimize AAV and scAAV vector payloads containing the Arg^UCA^ sup-tRNA. This powerful approach allows non-invasive biodistribution and longitudinal analysis by transcranial *in vivo* bioluminescence imaging (IVIS). These data show broad sup-tRNA tolerance and activity, highlighting the requirement of placing the tRNA within a native and short genomic sequence (100-bp) as well as the benefit of tRNA multiplexing. Strikingly, inclusion of tRNA introns increased scAAV titers by over 15-fold. Lastly, high-resolution tRNA sequencing performed on virally transduced brain samples reveals no consequence on native tRNA processing or expression, and shows that the anti-codon edited tRNA can achieve macroscopic rescue with expression levels at roughly ∼2% of the native parental Arg-TCT-1–1 tRNA. This *in vivo* vector-optimization strategy overcomes key barriers to clinical translation of sup-tRNA therapeutics for monogenic brain disorders.

## Materials and methods

### Reagents

gBlocks gene fragments synthesized by IDT; BamHI, KpnI, EcoRI, Monarch® Spin DNA Gel Extraction Kit (NEB, Cat. No. T1120), NEBuilder® HiFi DNA Assembly Master Mix (NEB, Cat. No. E2621), PolyJet transfection reagent (SignaGen Laboratories), Nano-Glo® Luciferase Assay System (Promega), D-Luciferin (GoldBio, Cat. No. eLUCK-1G; CAS No. 115144-35-9).

Specialized instruments used in this study included the Synergy Neo2 plate reader (BioTek), IVIS AMI imaging system (Spectral Instruments Imaging), Kopf neonatal stereotaxic frame, and Hamilton syringe (PN: 7635-01).

## Biological resources

### Cell lines

HEK293T cells were used for transient transfection-based rescue assays (293T; ATCC, Manassas, VA, USA; Cat. No. CRL-3216). HT1080 cells were used for AAV-based NanoLuc-TGA rescue assays provided by the UIOWA VVC.

### Mouse line

Heterozygous luciferase reporter mouse models (LumA) containing the R387X mutation (c.A1159T) in the luciferase gene located in the Rosa26 locus of the mouse genome were generated by breeding homozygous reporter mice (C57BL/6J-*Gt(ROSA)26Sor^em1Crx^*/J; The Jackson Laboratory, Bar Harbor, ME, USA; JAX Stock No. 038165) with WT mice C57BL/6J mice (The Jackson Laboratory, Bar Harbor, ME, USA; JAX Stock No. 000664; RRID:IMSR_JAX:000 664). This breeding strategy ensured that all offspring were heterozygous for the luminescent transgene. Genotyping was performed at postnatal day 21 (P21) to confirm heterozygosity. All animal procedures were approved by the Institutional Animal Care and Use Committee and were conducted in accordance with institutional and NIH guidelines.

### Plasmids and vectors

The G0463 Self-Complementary Shuttle Plasmid and G0463_pscAAV shuttle plasmid were obtained from the University of Iowa Viral Vector Core. The pUC19_mcs_P2a_NanoLUC vector, pcDNA3.1 vector, sup-tRNA-expressing plasmid, pscAAV_pCMV::mOrange2-Y156tga-BgHpA plasmid, pUC19_CDKL5_P2a_NanoLUC, pUC19_SYNGAP1_P2a_NanoLUC, pcDNA3.1::CDKL5_P2a_NanoLUC, and pcDNA3.1::SYNGAP1_P2a_NanoLUC were used as described below. Plasmid maps, full sequences, and primer sequences are provided in Supplementary Table S1.

The full-length CDKL5 sequence (RefSeq: NM_003 159, Clone ID: HsCD00022404) was obtained from the DNAsu repository at The Biodesign Institute, Arizona State University. The full-length SYNGAP1 sequence was kindly provided by Dr Gavin Rumbaugh (University of Florida).

### Viral vectors

AAV vectors were produced by Charles River Laboratories (251 Ballardvale Street, Wilmington, MA 01887, USA), PackGene Biotech (9310 Kirby Drive, Suite 900, Houston, TX 77054, USA), and the University of Iowa Viral Vector Core Facility, Carver College of Medicine (500 Newton Road, 221 Eckstein Medical Research Building, Iowa City, IA 52242, USA).

### Cloning and construct generation

#### Cloning of ArgUCA sup-tRNA constructs for virus production

Four gBlocks gene fragments, U6_tRNA_intron_Arg_TGA_ TCT_1–1, U6_tRNA_Arg_TGA_TCT 1–1, Geno_noI_tRNA-Arg_TGA_TCT_1–1, and Geno_I_tRNA-Arg_TGA_TCT_1–1 (Supplementary Table S1), were designed with overlapping sequences, synthesized by IDT, and cloned into the G0463 Self-Complementary Shuttle Plasmid from the University of Iowa Viral Vector Core after linearization using the BamHI restriction enzyme. The gBlocks and the linearized plasmid were purified using Monarch® Spin DNA Gel Extraction Kit (NEB, Cat. No. T1120). The assembly reactions were performed using the NEBuilder® HiFi DNA Assembly Master Mix (NEB, Cat. No. E2621) according to the manufacturer’s protocol. The assembled plasmids were then transformed into Stbl3 chemically competent *Escherichia coli* cells and plated on LB + carbenicillin selective media. Positive clones were confirmed by colony polymerase chain reaction (PCR) using LG153/154 primers (Supplementary Table S1) and by full plasmid sequencing at Plasmidsaurus.

The pCMV::mOrange2-Y156TGA insert was PCR amplified using LG160/LG262 primers (Supplementary Table S1) and cloned into the G0463_pscAAV shuttle plasmid at the BamHI restriction site using NEBuilder® HiFi DNA Assembly, resulting in the pscAAV_pCMV::mOrange2-Y156TGA-BgHpA plasmid. The four versions of tRNA-Arg-TCA-1–1 were PCR amplified using primers LG345/LG346 and LG206/207 and cloned into the pCMV::mOrange2-Y156TGA-BgHpA plasmid at the SphI restriction site using NEBuilder® HiFi DNA Assembly.

### Cloning of SYNGAP1/CDKL5 wild-type and Arg CGA-to-TGA mutants

WT coding sequences (CDS) of CDKL5 and SYNGAP1 were PCR-amplified using primers LG967/969 or LG935/937, respectively, and cloned into the pUC19_mcs_P2a_NanoLUC vector linearized with KpnI using NEBuilder® HiFi DNA Assembly, resulting in the constructs pUC19_CDKL5_P2a_NanoLUC and pUC19_SYNGAP1_P2a_NanoLUC. To generate the Arg-to-TGA mutants, the mutant sequences were PCR-amplified using primer sets LG964/LG966/LG974–LG987 for CDKL5 or LG964/LG966/LG1008–LG1038 for SYNGAP1. These were cloned into pcDNA3.1 linearized with BamHI and EcoRI using NEBuilder® HiFi DNA Assembly, yielding pcDNA3.1::CDKL5_P2a_NanoLUC and pcDNA3.1::SYNGAP1_P2a_NanoLUC.

### *In vitro* rescue assays

#### PTC rescue in *SYNGAP1* and *CDKL5 in vitro*

HEK293T cells were seeded in six-well plates and cultured to ~80% confluency. Cells were then transiently transfected with 0.5 µg of either pcDNA3.1::CDKL5_WT/TGAmut_P2a_NanoLUC or pcDNA3.1::SYNGAP1_WT/TGAmut_P2a_NanoLUC, along with 0.5 µg of sup-tRNA-expressing plasmid, using PolyJet transfection reagent (SignaGen Laboratories). Twenty-four hours post-transfection, cells were lysed, and NanoLuciferase activity was measured using the Nano-Glo® Luciferase Assay System (Promega) on a Synergy Neo2 plate reader (BioTek).

### *In vitro* NanoLuc-TGA rescue

HT1080 cells were seeded in 96-well plates and transduced with the indicated sup-tRNA AAV vectors. At ~80% confluency, cells were transfected 6 h post-transduction with a NanoLuciferase reporter containing an in-frame TGA PTC (NanoLuc-TGA) using PolyJet transfection reagent, according to the manufacturer’s instructions. Luminescence was measured at 24 and 48 h post-transduction using the Nano-Glo® Luciferase Assay System (Promega) and read on a Synergy Neo2 microplate reader (BioTek). Signal was quantified as relative luminescence units for each well and analyzed as specified in Supplementary Table S2.

### Intracerebroventricular injections

Intracerebroventricular (ICV) injections were performed on postnatal day 0–1 (P0–P1) mouse pups. Neonates were anesthetized by hypothermia by placement on ice for 3 min, in accordance with institutional animal care and use guidelines. Pups were secured in a Kopf neonatal stereotaxic frame, and the head was stabilized for injection. Using a 32-gauge needle attached to a Hamilton syringe (PN: 7635-01), the specified dose of ss/scAAV2/9 Arg^UCA^ sup-tRNA vector was delivered into the lateral ventricle using the following coordinates: *X* = 0.8 mm, *Y* = 1.5 mm, *Z* = 1.5 mm.

### Bioluminescence imaging

#### *In vivo* bioluminescence imaging

To assess *in vivo* brain luminescence, postnatal day 30 (P30) mice were imaged using the IVIS AMI imaging system (Advanced Molecular Imager, Spectral Instruments Imaging). D-Luciferin (GoldBio, Cat. No. eLUCK-1G; CAS No. 115144-35-9) was administered intraperitoneally at 150 mg/kg body weight. Following injection, mice were returned to their chamber and maintained under isoflurane anesthesia until imaging. A 20-min interval was allowed to ensure sufficient penetration of luciferin across the blood–brain barrier. Imaging was performed with a 60-s exposure time and a field of view set to 20 cm. Bioluminescent signal was quantified using AURA *In Vivo* Imaging Software (Spectral Instruments Imaging) by measuring photon flux within the region of interest (ROI) encompassing the brain.

### *Ex vivo* bioluminescence imaging

For *ex vivo* analysis, mice were euthanized under isoflurane anesthesia, and brains were rapidly harvested and placed in small dishes. A total of 200 µl of D-Luciferin solution was evenly pipetted over both hemispheres of each brain to ensure full substrate coverage. Brains were imaged using the IVIS AMI system with *ex vivo* acquisition settings using a 60-s exposure. Luminescence was quantified by ROI-based photon flux measurements using AURA *In Vivo* Imaging Software.

### Brain section staining and fluorescence imaging

P30 mice were transcardially perfused with phosphate buffer, followed by 4% paraformaldehyde (PFA). Brains were harvested, post-fixed in 4% PFA for 24 h, cryoprotected sequentially in 30%, 20%, and 10% sucrose for 24 h each, embedded in optimal cutting temperature compound, and sectioned on a cryotome at 20-µm thickness. For endogenous mOrange2 fluorescence imaging, sections were coverslipped with Fluoromount-G with DAPI (Invitrogen/Thermo Fisher Scientific, Cat. No. 00-4959-52). Images were acquired on a Leica SP5 confocal microscope with an HC PL Fluotar 20×/0.55 NA dry objective. DAPI was acquired using a 405-nm laser at 1% power with a 410–480-nm detection window, and mOrange2 fluorescence was acquired using a 552-nm laser at 5% power with a 575–670-nm detection window. For cleaved caspase-3 immunohistochemistry, sections were rinsed three times in Dulbecco’s phosphate-buffered saline (DPBS), blocked/permeabilized in 5% normal donkey serum (Millipore Sigma, Cat. No. S30-M) with 0.3% Triton X-100, and incubated overnight at 4°C with rabbit monoclonal anti-cleaved caspase-3 antibody (Asp175; clone 5A1E; Cell Signaling Technology, Danvers, MA, USA; Cat. No. 9664; 1:400). Sections were rinsed three times in DPBS, incubated for 2 h at room temperature with chicken anti-rabbit IgG (H + L) cross-adsorbed Alexa Fluor 488 secondary antibody (Thermo Fisher Scientific, Waltham, MA, USA; Cat. No. A-21441; 1:1000), rinsed three times in DPBS, and coverslipped with Fluoromount-G with DAPI. DAPI was acquired using a 405-nm laser at 1% power with a 410–480-nm detection window, and cleaved caspase-3 immunostaining was acquired using a 488-nm laser at 5% power with a 495–570-nm detection window.

### tRNA sequencing and bioinformatic analysis

Analysis of tRNA abundance, processing, and acylation was performed by MesoRNA (Chicago, IL, USA). Paired-end reads were split by barcode sequence using Je demultiplex with the options “BPOS = BOTH BM = READ_1 LEN = 4:6 FORCE = true C = false.” FASTQ files from individual samples were then mapped against a reference genome of mouse mature nuclear-encoded tRNAs, with Arg-TCA-1–1 added, and intron-containing variants of Arg-TCA-1-1 and native Arg-TCT-1-1. Alignments were performed using read 2 only with Bowtie2 in local alignment mode. Mapped reads were then filtered using a custom script to include only reads where one end is 5′ of position 32 and the other end is 3′ of position 37, thus ensuring coverage across the Arg^UCU/^Arg^UCA^ anticodon and eliminating ambiguous reads. tRNA abundance measurements were derived from counting the number of filtered reads mapping to each reference gene. For modification analysis, SAM files were converted to WIG files using IGV-2.19.6 count command with the options “-z 5 -w 1 -e 250 –bases.” WIG files were then reformatted using custom scripts from the original MSR-seq pipeline available as Supplementary Data. Data used for graphing are provided in Supplementary Table S3.

### Statistical analyses

Data are presented as mean ± SEM unless otherwise indicated. Statistical significance was defined as *P* < .05 after correction for multiple comparisons where applicable. Statistical analyses and sample sizes for each experiment are provided in Supplementary Table S2.

Two-group comparisons were analyzed using unpaired two-tailed *t*-tests, with Welch’s correction when variances were unequal. Multiple two-group comparisons, including the SYNGAP1 and CDKL5 *in vitro* rescue assays, were corrected using the Holm–Šídák method; these assays used *n* = 4 independent measurements per condition for each construct.

Comparisons involving more than two groups were analyzed using Brown–Forsythe and Welch analysis of variance (ANOVA) followed by Dunnett’s T3 multiple-comparisons test. This approach was used for multi-group luminescence comparisons *in vitro* and *in vivo*. The HT1080 NanoLuc-TGA AAV rescue assay used *n* = 3 technical replicate wells per group/time point, and *in vivo* group sizes are provided in Supplementary Table S2.

*Ex vivo* brain bioluminescence was analyzed using an unpaired two-tailed *t*-test with Welch’s correction (*n* = 4 per condition). Longitudinal *in vivo* bioluminescence was analyzed using two-way ANOVA followed by Šídák’s multiple-comparisons test (*n* = 3–4 per condition).

For tRNA sequencing analyses, uninjected and injected groups were compared using multiple unpaired *t*-tests with Welch’s correction or Welch ANOVA, as specified in Supplementary Table S2. Abundance values were transformed as *Y* = log(*Y* + 0.000001), and analyses were performed with *n* = 3 per group where both comparison groups were present.

### Novel programs, software, and algorithms

Software and computational tools included GraphPad Prism for statistical analyses, AURA *In Vivo* Imaging Software for bioluminescence quantification, LAS X Navigator for confocal mosaic acquisition, Je demultiplex for barcode demultiplexing, Bowtie2 for sequence alignment, IGV-2.19.6 for WIG file generation, and custom scripts from the MSR-seq pipeline for tRNA-seq processing. Figure schematics and icons were created using BioRender.

### Web sites and database referencing

tRNA sequencing data will be available on NCBI Gene Expression Omnibus.

## Results

### Assessing PTC burden in brain and agnostic rescue in SYNGAP1 and CDKL5

PTCs are a major driver of neurodevelopmental and neurological disorders, Fig. 1A and B. An analysis from the clinical variant database ClinVar shows that nonsense variants account for roughly 10%–45% of all patients in the 11 genes that were assessed. The highest burdens were observed in *CHD2* (42%) and *SYNGAP1* (35%), followed by *CACNA1A, FOXG1*, and *PCDH19* (23%). Across all genes, Arg CGA-to-TGA PTCs were the most common variants, reaching the highest abundances in *SYNGAP1* (54%), *SCN2A* (51%), *SCN1A* (43%), and *CHD2* (42%). A second prominent gene-dependent enrichment involved Gln-to-TAG variants. This class was especially frequent in *SLC6A1* (46%) and *SLC2A1* (41%), and was also present in *CDKL5* (34%) and *FOXG1* (27%), indicating that in these genes a large fraction of nonsense variants arises from Gln-to-TAG substitutions.

Overall, the distribution of nonsense variants is dominated by a small number of recurrent sequence contexts, particularly Arg-to-TGA, with additional gene-specific enrichment for Gln-to-TAG and a limited set of secondary contexts. The submission summary by PTC type showed a similarly skewed pattern, Fig 1B. Arg-to-TGA accounted for 34% of submissions and Gln-to-TAG accounted for 17%. Each remaining context contributed 8% or less, forming a long tail of lower-frequency events. Because arginine CGA-to-TGA variants represented the most frequent class of PTCs, we examined the local nucleotide composition surrounding patient-derived CGA-to-TGA sites. Analysis of a 67-bp window centered on the mutant TGA codon among the 19 variants showed modest positional differences in nucleotide frequency near the mutated codon, Supplementary Fig. S1. These observations suggest modest positional bias, with cytosine enriched immediately 5′ of CGA (52.6%) and purines enriched at the +1 position (G, 47.4%; A, 42.1%). However, further analysis will be required to determine whether specific flanking nucleotides influence suppression efficiency.

### Sup-tRNA rescues translation across patient-derived UGA sites with variable efficiency

To test whether the Arg^UCA^ sup-tRNA supports unbiased suppression across patient-derived TGA sites, we generated complementary DNA constructs corresponding to each Arg-TGA mutation in *SYNGAP1* and *CDKL5*, and fused them to a P2A-NanoLuciferase reporter, Fig. 1C. Constructs were transfected into HEK293T cells either without sup-tRNA (-sup-tRNA) or with a sup-tRNA-Arg-TCA-1–1 expression plasmid (+sup-tRNA), and luminescence was quantified by plate reader 24 h post transfection.

While all *SYNGAP1-*TGA mutants showed markedly reduced signal without sup-tRNA (0.7%–3.7% of WT), co-expression of Arg^UCA^ tRNA increased luminescence for every mutant (37%–104% of WT), Fig. 1D. This corresponds to 28- to 82-fold increases relative to the -sup-tRNA condition. Additionally, rescue varied modestly across sites, with the lowest mean signal at R105X (1.21 × 10^6^ a.u.) and near-WT recovery for R76X and R526X (3.25–3.39 × 10^6^ a.u.). Consistent with previous results, *CDKL5-*TGA mutants were reduced without sup-tRNA (0.6%–14% of WT), while co-transfection of the sup-tRNA increased luminescence across all sites, Fig. 1E. Rescue was weakest at R134X (8.2% of WT), whereas multiple sites (e.g. R59X, R550X, R559X, R970X, and R981X) recovered to approximately WT levels. Together, these data show broad suppression across patient-derived TGA sites, with measurable variability between individual mutations.

### Assessing sup-tRNA performance *in vitro*

**Figure 2.**
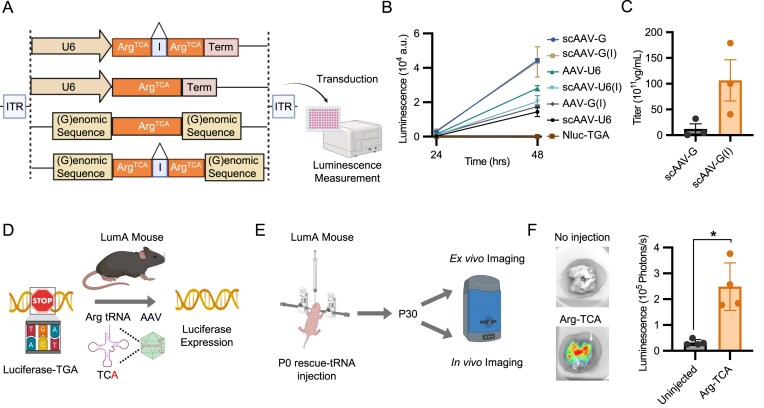
Payload optimization and *in vivo* assessment. (**A**) Schematic of sup-tRNA expression cassettes packaged into ssAAV and scAAV vectors. Constructs are U6-driven or genomically flanked, each including or excluding an intron sequence within the tRNA-Arg-TCT1-1. Vectors were used to transduce NanoLuc-TGA reporter HT1080 cells. (**B**) Luminescence of transduced cells measured at 24 and 48 h post-transduction, demonstrating *in vitro* PTC suppression efficiency of each construct (*n* = 3 per construct). (**C**) Viral titers of scAAV-G and scAAV-G(I) measured by qPCR (*n* = 3). (**D, E**) *In vivo* delivery of sup-tRNA constructs into neonatal LumA mice via ICV injection. (**F**) *Ex vivo* luminescence measurement of injected versus uninjected LumA brains confirming localized rescue (*P* < .05, unpaired *t*-test with Welch’s correction test). Created in BioRender. Al saneh, A. (2026) https://BioRender.com/2ce96kj.

Given the potential for an Arg^UCA^ sup-tRNA to rescue PTCs in HEK cells, we aimed to develop a payload that would be successful for delivery to mammalian brain. Recent efforts have made modest improvements in payload designs [22, 27]; however, *in vivo* tRNA potency remains a challenge. We therefore tested multiple tRNA flanking sequences for their ability to improve viral vector titers as well as potent payloads with *in vivo* activity. The variables that we focused on were traditional single-stranded AAV and scAAV, as the latter has a smaller double-stranded payloads that we hypothesized to be more tolerant of tRNA. We also examined the standard U6 RNA promoter compared to the short native genomic context surrounding the tRNA within its genomic context. Here, we reasoned that the genomic, and potentially the short tRNA intronic, context may provide more efficient expression and tRNA processing. To evaluate the capacity of Arg^UCA^ sup-tRNA constructs to rescue PTCs, we generated vectors encoding the sup-tRNA with or without an intronic sequence, driven by either its native genomic context or an upstream U6 promoter. Constructs were packaged into AAV or scAAV and delivered to HT1080 cells transiently expressing a Nano-Luciferase reporter containing a premature TGA stop codon (NLuc-TGA), Fig. 2A. We then quantified reporter rescue using a luminescence-based plate reader assay, Fig. 2B.

At 24 h post-transduction, all sup-tRNA configurations produced measurable increases in luminescence signal relative to NLuc-TGA controls, although with marked differences in magnitude. Specifically, constructs within a 100-bp genomic context, both with the tRNA intron (scAAV-G(I)) and without the intron (scAAV-G), produced the highest signals, followed by AAV-U6 and AAV-G(I), Fig. 2B. By 48 h, luminescence signals increased substantially across all conditions, indicating continued accumulation of functional reporter protein over time. scAAV-G and scAAV-G(I) remained the most potent configurations, while intermediate signals were observed for AAV-U6, scAAV-U6(I), AAV-G(I), and scAAV-U6; the NanoLuc-UGA reference remained low. These results demonstrate that the Arg^UCA^ sup-tRNA within a genomic context using scAAV outperforms other configurations *in vitro*. Interestingly, across three independent production sources (University of Iowa Viral Vector Core; Charles River; PackGene), scAAV intron-containing vectors were consistently higher in titers than those purely within genomic context, Fig. 2C. The inclusion of the intron may delay the accumulation of mature sup-tRNA, thus reducing interference with AAV packaging functions.

### Self-complementary AAV enables superior delivery of sup-tRNA in mammalian brain

To assess whether Arg^UCA^ sup-tRNA-mediated rescue could be detected in the brain, we utilized a reporter mouse line that carries a luciferase gene containing an p.R387X (TGA) premature stop codon at the *Rosa26* locus [28]. In this model, luciferase activity provides a direct readout of UGA stop-codon suppression, Fig 2D. LumA reporter mice were first injected ICV at postnatal day 0 (P0), and brains were harvested at P30 for *ex vivo* bioluminescence imaging using IVIS, Fig 2E. scAAV-G(I) brains exhibited a 7.7-fold increase in signal, indicating sustained reporter rescue in the brain, Fig. 2F and Supplementary Fig. S2.

**Figure 3.**
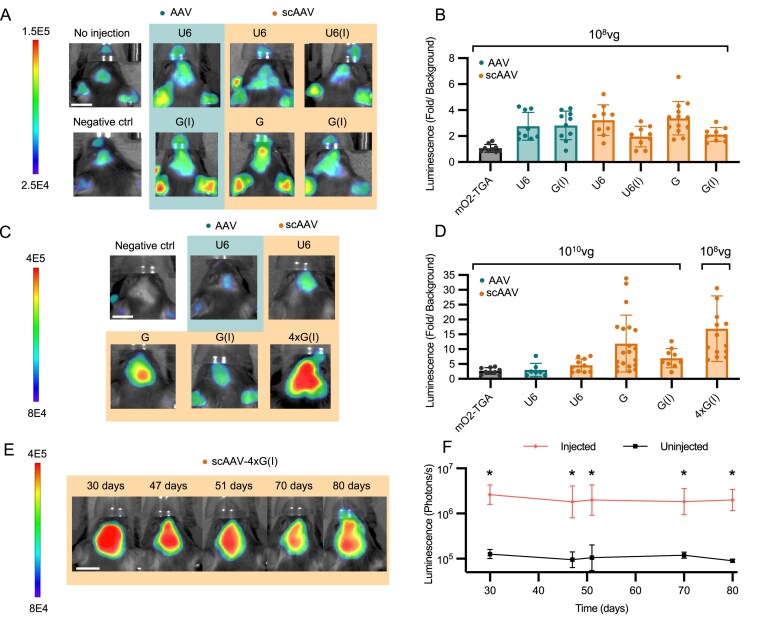
Potent and durable UGA PTC rescue in brain with scAAV and minimal genomic context. (**A**) Representative transcranial IVIS images and quantification of LumA mice injected with a low dose of sup-tRNA constructs (7 × 10^8^ vg). (**B**) Luminescence rescue (fold/background) for each injected condition at 7 × 10^8^ vg. Scale bar: 1 cm. (**C, D**) Genomic context provides highest rescue, and multiplexing to 4× tRNA copies enables similar rescue at 14-fold lower viral dose of 7 × 10^8^ vg. (**E, F**) Longitudinal IVIS imaging demonstrating durable nonsense suppression following scAAV-4xG(I) delivery over 30–80 days post-injection (*n *= 3 per condition). Data are shown as mean ± SD with individual biological replicates.

After confirming activity of the Arg^UCA^ sup-tRNA in mouse brains, neonatal (P0–P1) LumA mice received ICV injections of AAV9 or scAAV9 vectors within the different configurations made, Fig 3. At P30, *in vivo* bioluminescence was quantified by IVIS in live anesthetized mice as ROI luminescence and normalized to background luminescence in the LumA mouse line. We used a scAAV-mO2-TGA construct as a negative control for this set of experiments. This construct had a CMV-driven mO2 payload with a TGA stop codon inserted at Y156. We have found this position on mO2 to be permissive of encoding a variety of amino acids, and its structural location on the outside of the protein is consistent with tolerance of missense variants. This overall approach unlocks a direct comparison of *in vivo* rescue for payload assessment in mammalian brain. In combination with live animal IVIS transcranial imaging, the rescue of the LumA luciferase element can be non-invasively measured and tracked over the lifetime of the animal. At a dose of 7 × 10^8^ vg per mouse, the mO2-TGA negative control vector showed no increase above baseline luminescence, Fig. 3A and B. In contrast, five out of the six tRNA-expressing vectors produced significant reporter rescue when compared to the negative control. The strongest signals were observed for scAAV-G and scAAV-U6, each producing ∼3.3-fold increase above background. Constructs containing an intron exhibited more moderate effects, with scAAV-G(I) and scAAV-U6(I) displaying a two-fold increase in luminescence while single-stranded AAV vectors yielded intermediate rescue of ∼2.8-folds over background. The additional processing steps required by the intron-containing constructs to reach their mature form could be rate-limiting, thereby imposing a modest cost on per-particle potency.

Increasing the dose to 1 × 10^10^ vg further enhanced reporter rescue, Fig. 3C and D. While the scAAV-mO2-TGA negative control showed a modest signal in luminescence, self-complementary tRNA-expressing vectors with the genomic sequences produced substantially higher luminescence levels. Relative to this control, scAAV-G produced the largest increase (12-fold over background), followed by scAAV-G(I) (seven-fold over background). Meanwhile, scAAV-U6 and AAV-U6 showed no significant differences than the negative control at the tested dose, yielding 4.5-fold and 2.9-fold increases over background, respectively. Notably, at the lower dose of 7 × 10^8^ vg per mouse, increasing the copy number of the tRNA to four, while keeping the genomic and intron sequences, outperformed the higher dose of 1 × 10^10^ vg per mouse. scAAV-4×G(I) yielded over 16-fold over background, exceeding the mean signal of all high-dose constructs in this dataset, Fig. 3C and D. Thus, the small size of the tRNA in the payload opens up the possibility of tRNA multiplexing and lower viral doses.

To assess sup-tRNA-mediated rescue of a PTC reporter *in vivo*, we examined native mOrange fluorescence in coronal brain sections following scAAV-G(I) delivery of the mO2-TGA reporter with or without 1× Arg^UCA^ sup-tRNA, Supplementary Fig. S3. In sections from mice receiving the mO2-TGA reporter alone, native mOrange signal was undetectable, consistent with termination at the introduced premature TGA codon. In contrast, co-expression of mO2-TGA and Arg^UCA^ tRNA produced widespread mOrange-positive neurons across the injected brain region. Additionally, we assessed potential toxicity by performing cleaved caspase-3 immunohistochemistry on brain sections from scAAV-G-injected mice. Cleaved caspase-3 staining was not detectable, thus indicating that the Arg^UCA^ sup-tRNA is broadly distributed throughout the examined brain region, and does not induce detectable caspase-3-associated toxicity.

### scAAV9 tRNA delivery provides durable rescue

To determine whether tRNA-mediated rescue persists over time *in vivo*, we next assessed the durability of the most effective tRNA construct, scAAV-4×G(I), using longitudinal bioluminescence imaging in LumA mice, Fig. 3 E and F. Following ICV neonatal injections, reporter signal was quantified between postnatal day 30 (P30) and P80 and compared to age-matched uninjected controls. Luminescence in injected mice remained consistently elevated, showing a 17.25–24.17-fold difference between groups across time points.

### *In vivo* profiling of arginine tRNA isodecoder abundance, charging, and processing

**Figure 4.**
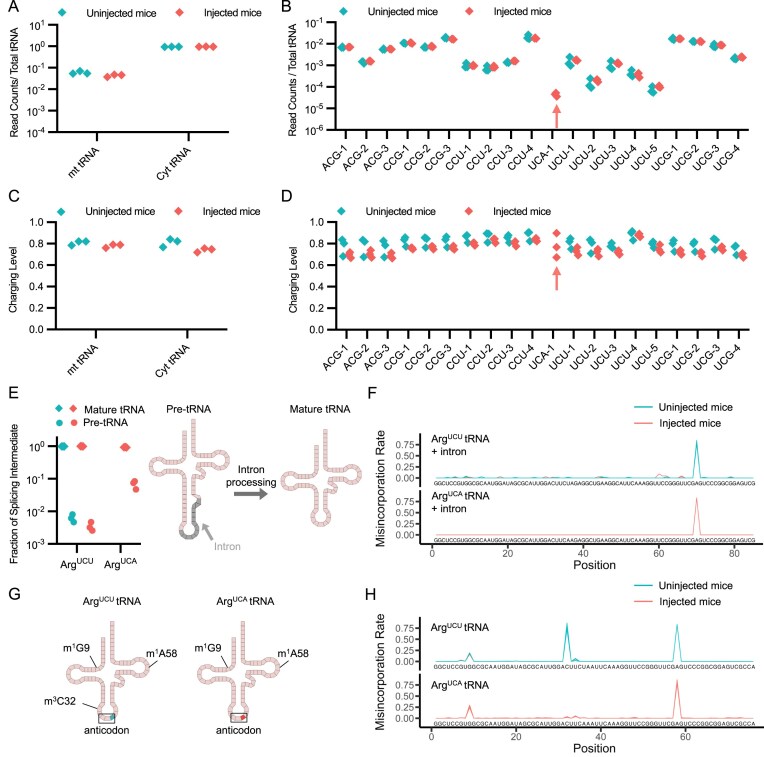
Brain tRNA sequencing shows preserved endogenous tRNA abundance, aminoacylation, and processing following sup-tRNA delivery. (**A**) Global tRNA abundance in uninjected and sup-tRNA-injected mouse brains, separated into mitochondrial (mt) and cytosolic (Cyt) tRNAs. (**B**) tRNA-Arg isodecoder-level abundance in both conditions, showing detection of Arg^UCA^ tRNA only in injected brains with no apparent changes across endogenous Arg-tRNAs. (**C**) Aminoacylation levels of mt and Cyt tRNA. (**D**) Charging levels of tRNA-Arg isodecoders confirm charging of Arg^UCA^ tRNA in injected brains. (**E**) Fraction of mature versus premature parental and sup-tRNAs in uninjected and injected brains. (**F**) Misincorporation profiles across intron-containing Arg^UCU^ and Arg^UCA^ pre-tRNAs in uninjected and injected mice, plotted by nucleotide position. Peaks indicate positions with elevated reverse-transcription misincorporation, consistent with modified nucleotides retained in pre-tRNA species. (**G**) Schematic structures indicating annotated modification sites: m^1^G9 and m^1^A58 in both Arg^UCU^ and Arg^UCA^ tRNAs, and m^3^C32 in Arg^UCU^ tRNA. (**H**) Nucleotide-resolution misincorporation profiles across mature Arg^UCU^ and Arg^UCA^ tRNAs from uninjected and injected mice (*n* = 3). Created in BioRender. Al saneh, A. (2026) https://BioRender.com/2ce96kj.

There are 28 human arginine tRNA genes, and it is possible that viral expression of a sup-tRNA may alter the expression balance and/or tRNA acylation efficiency. Indeed, all Arg tRNA are aminoacylated by a single aminoacyl-tRNA synthetase (RARS). Thus, the unintended effect of Arg^UCA^ sup-tRNA expression could be the competition for RARS activity. To determine whether neonatal scAAV-G(I) delivery alters the endogenous tRNA landscape in brain, and to confirm expression and aminoacylation of the Arg^UCA^ sup-tRNA, ICV-injected LumA mouse brains were harvested at P30, and tRNA were extracted for full sequencing and analysis. By analyzing the total mitochondrial (mt) and cytosolic (Cyt) tRNA read counts, sequencing data revealed that both injected and uninjected brains expressed similar abundances: cytosolic tRNAs comprised 95.6% and 94.1% of the total tRNA, respectively, while mitochondrial tRNAs accounted for 4.4% and 5.9%, Fig. 4A.

Within the cytosolic Arg tRNA isodecoder dataset, all species showed similar fractional abundances between the two groups, with the sup-tRNA readily detected in the injected mouse brains and comprising only 1.2% of the Arg^UCU^ tRNA anticodon family, Fig. 4B.

Additionally, the charging fractions for the global tRNAs as well as the Arg tRNAs in both groups were not different, with Arg^UCA^ tRNA ranking right above the median, Fig. 4C and D. To confirm that the sup-tRNA undergoes canonical maturation *in vivo*, we quantified the relative abundance of mature versus intron-containing (premature) tRNA species in injected mouse brains. For parental Arg^UCU^ tRNA, the mature fraction was near 100% in both uninjected and injected brains, consistent with robust endogenous processing, Fig. 4E. Similarly, Arg^UCA^ tRNA was post-translationally processed in the injected mice, as indicated by the predominance of the mature tRNA species following intracranial delivery.

We next compared the modification profiles of the Arg^UCA^ sup-tRNA and the native Arg^UCU^ tRNA by quantifying misincorporation rates at each nucleotide position Fig. 4F–H. These rates provide an indirect readout of endogenous modification sites, because certain tRNA modifications, such as methylation, disrupt canonical base pairing during reverse transcription, leading to incorrect nucleotide incorporation. In both injected and uninjected brains, peaks were observed at positions corresponding to m^1^G9 and m^1^A58 for both Arg tRNA species. In contrast, a signature at position 32, indicative of m^3^C32, was absent in Arg^UCA^ sup-tRNA. This is consistent with METTL2A/2B-mediated recognition of position 36 [29]. Therefore, the delivered Arg^UCA^ sup-tRNA is detected by tRNA-seq and processed *in vivo* as a bona fide tRNA substrate in brain, while retaining an anticodon-dependent modification profile consistent with its engineered identity. Further, the overall expression of endogenous tRNA is similarly not affected by the expressed Arg^UCA^ sup-tRNA, Supplementary Fig. S4. In terms of off-target consequences of the expressed Arg^UCA^ sup-tRNA, the tRNA sequencing suggest that the expressed tRNA is processed similarly to endogenous Arg tRNAs and that its expression does not measurably perturb the processing or aminoacylation of endogenous Arg tRNA isodecoders.

## Discussion

Here we describe a programmable method using human sup-tRNA which enables a general strategy to rescue UGA PTCs in mammalian brain. A primary concern of any read-through strategy is the potential interactions with native stop codons, which terminate translation. For instance, small-molecules read-through agents (e.g. Ataluren) promote mis-encoding at the site of the PTC as well as elsewhere in the proteome [12], yet are deemed safe for clinical use [30]. By contrast, sup-tRNA encode the correct amino acid at the PTC—a key feature for proteins with low tolerance for missense variants—and appear to have minimal interactions with native stops [18, 24, 31]. Possible mechanisms for this observation have been previously summarized [17] and suggest that engineered tRNA display a “stop codon” bias for PTC variants over native 3′ stops due to the genetic context of native stop codons [32, 33]. There are likely many unresolved details in this mechanism, and additional work is required to understand how this ribo-protein complex works in concert to minimize sup-tRNA read-through. An additional challenge for the development of a tRNA-based therapy is the possibility that expression of a sup-tRNA could alter the processing or expression of other tRNAs from the same family (e.g. Arginine). The data generated here from virally delivered tRNA in brain suggest that, at the doses delivered, tRNA acylation, methylation, and expression are unchanged. However, future studies will be needed to better understand the tRNA expression threshold of these observations. Taken together, these data show that there is a therapeutic window of sup-tRNA expression that promotes PTC rescue *in vivo*, yet does not affect native stop codons or tRNA processing.

With this apparent safety profile in mind, we examined tRNA-bearing payload designs in the brains of P0 mice using ICV injection of viral vectors. The reason for this experimental approach was twofold. First, many of the neurodevelopmental disorders shown in Fig. 1 would benefit from early intervention, as it is unknown if there are therapeutic windows where a given rescue strategy will be most effective. It is reasonable to believe that earlier treatments are, generally, better [34, 35]. Second, the neurodevelopmental changes in the mammalian brain between birth and adolescence are marked by extreme molecular, cellular, and structural growth, making this an especially fragile and fraught period for gene regulation. By delivering our most potent tRNA payload, a 4× Arg^UCA^ sup-tRNA based on the natural human tRNA with highest activity, this approach serves as a tolerance test of the method, as the tRNA would be present during the many crucial milestones of brain development. Notably, we observed no obvious changes to mouse behavior or physiology, although additional studies will be needed to expand this analysis to subregions of the brain and to perform more detailed behavioral assessments. Consistent with this possibility, we find no evidence of the detrimental neurological marker of caspase-3 activation after delivery of a 1× sup-tRNA payload demonstrated broad rescue, evidenced by recovered mO2 fluorescence in cortical slices, Supplementary Fig. S3.

In conclusion, this work provides a safety threshold for sup-tRNA *in vivo*, a means for efficient viral production and assessment of biological activity with the LumA mouse model, and methods to quantify off-target interactions. Given the unmet need from the many currently untreated genetic diseases caused by PTC variants, this study brings the possibility of a first-in-class therapeutic tRNA strategy one step closer to the clinic.

## Supplementary Material

gkag695_Supplemental_Files

## Data Availability

The tRNA sequencing data have been deposited in GEO (https://www.ncbi.nlm.nih.gov/geo/) under accession number GSE334212. Custom scripts are available in the Supplementary Data. All relevant data are included within the article and its Supplementary Information files. Requests for further information and resources should be directed to and will be fulfilled by the lead contact, Christopher A. Ahern (E-mail: christopher-ahern@uiowa.edu).
