# Supplementary material for: Programmed repair of disease-causing UGA premature termination codons in mammalian brain
Source: Nucleic Acids Res. 2026 Jul 16;54(13):gkag695. doi: 10.1093/nar/gkag695 (PMC13373322; doi:10.1093/nar/gkag695)
Supplement: gkag695_Supplemental_Files [file gkag695_supplemental_files.zip › Supplementary_Figures_v3.pdf]

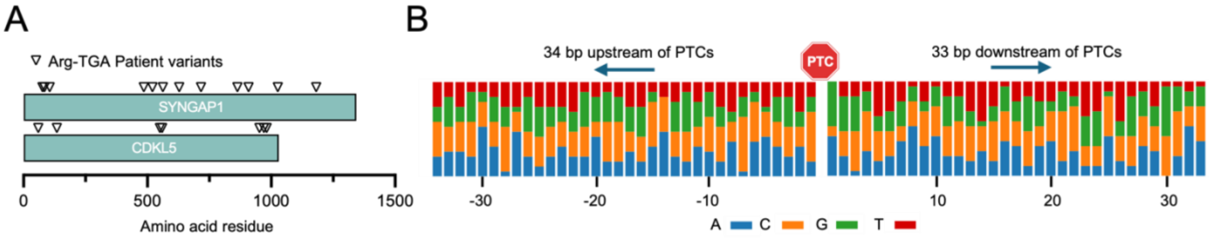

Figure S1. Patient-derived arginine-to-UGA nonsense variants selected to assess whether sequence context affects suppressor tRNA-mediated rescue efficiency. A. patient-derived CGA to UGA variants discovered in *SYNGAP1* (R76UGA, R84UGA, R105UGA, R485UGA, R520UGA, R526UGA, R628UGA, R716UGA, R863UGA, R908UGA, R1026UGA and R1181UGA) and *CDKL5* (R59UGA, R134UGA, R550UGA, R559UGA, R952UGA, R970UGA and R981UGA). B. Nucleotide composition across a 67-nt window centered on the mutant UGA premature termination codon (34 bp upstream and 33 bp downstream) across all 19 variants. Flanking sequence context shows modest positional bias, with cytosine enriched immediately 5′ of CGA (52.6%) and purines enriched at the +1 position (G, 47.4%; A, 42.1%). Stacked bars indicate the nucleotide frequency at each position.

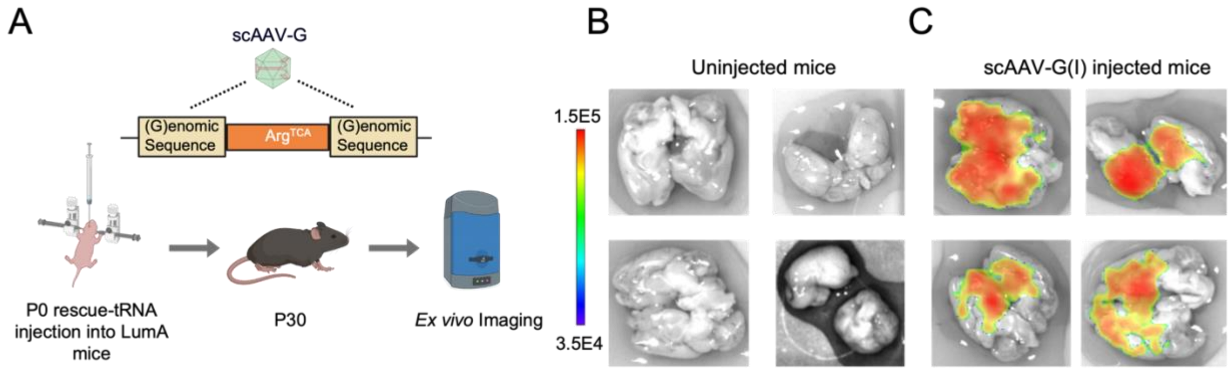

Figure S2 Ex-vivo bioluminescence imaging of LumA mouse brain shows rescue of Firefly Luciferase-TGA via suppressor tRNA. A. Postnatal day 0 (P0) neonatal intracerebroventricular injection delivering one copy of Arg^{UCA} tRNA flanked with genomic sequences in scAAV2/9 (scAAV-G) at the dose of $1{\times}10^{10}$ vg, followed by ex vivo imaging at P30. **B.** Ex vivo brain luminescence imaging from uninjected LumA mice show absence of detectable signal. **C.** Ex vivo brain luminescence imaging from scAAV-G-injected LumA mice show robust reporter signal, indicated by pseudocolor overlay, demonstrating suppressor tRNA-dependent rescue of luciferase-TGA of LumA activity. Created in BioRender. Al saneh, A. (2026) https://BioRender.com/2ce96kj

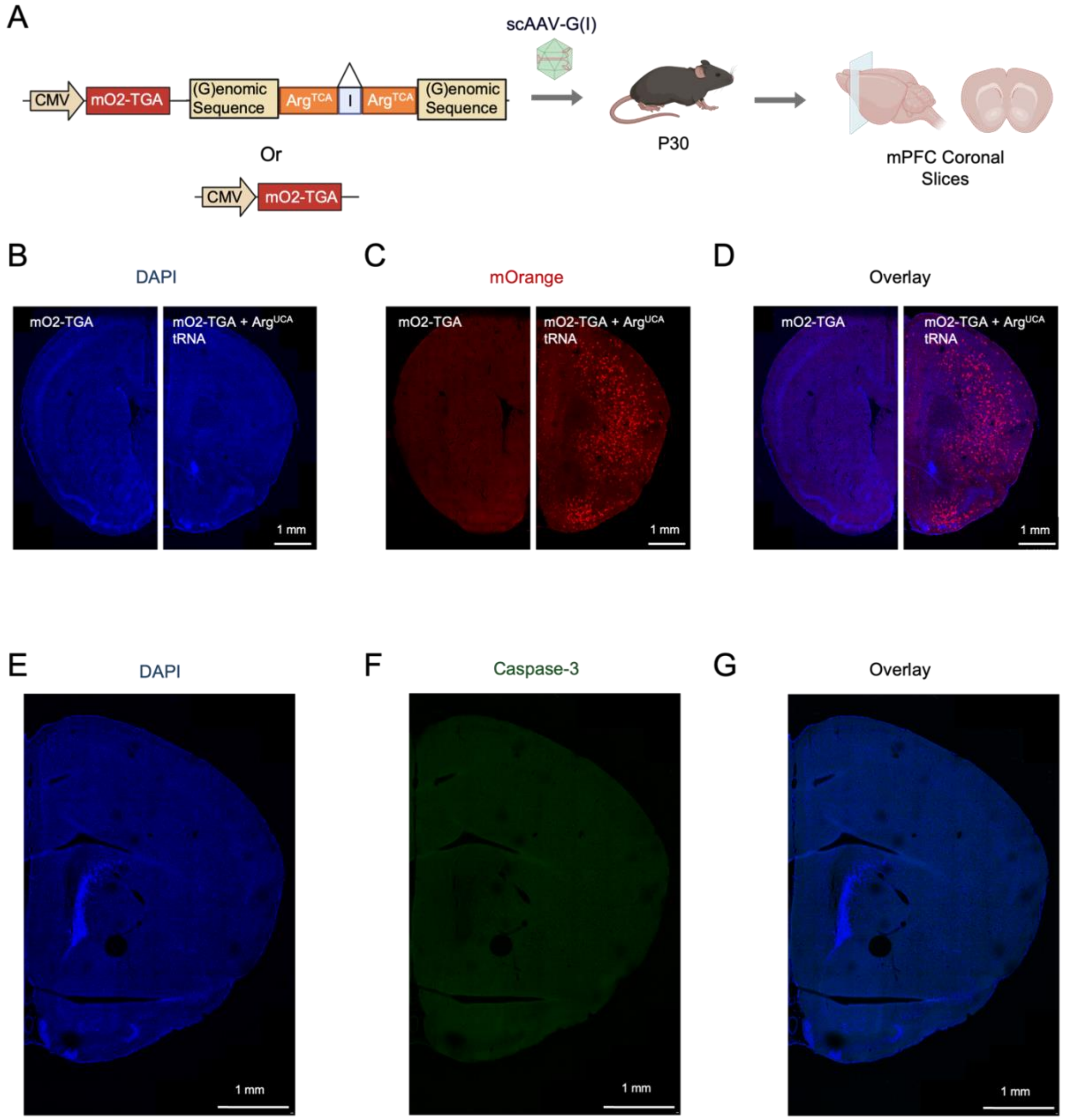

Figure S3 Suppressor tRNA-dependent mOrange fluorescence and IHC-based toxicity assessment in mouse brain. A. Schematic of scAAV2/9 delivery of the mO2-TGA reporter alone or with Arg^{UCA} suppressor tRNA (scAAV-G(I)), followed by analysis of P30 medial prefrontal cortex (mPFC) coronal sections. **B–D**, Representative coronal brain sections showing DAPI, native mOrange fluorescence, and merged signal. mOrange fluorescence was not detected with mO2-TGA alone but was restored by co-expression of Arg^{UCA} tRNA. **E–G,** Representative sections stained for DAPI and caspase-3. Absent caspase-3 signal indicates no evident caspase activation or apoptotic toxicity following suppressor tRNA delivery. Scale bars, 1 mm. Created in BioRender. Al saneh, A. (2026) https://BioRender.com/2ce96kj

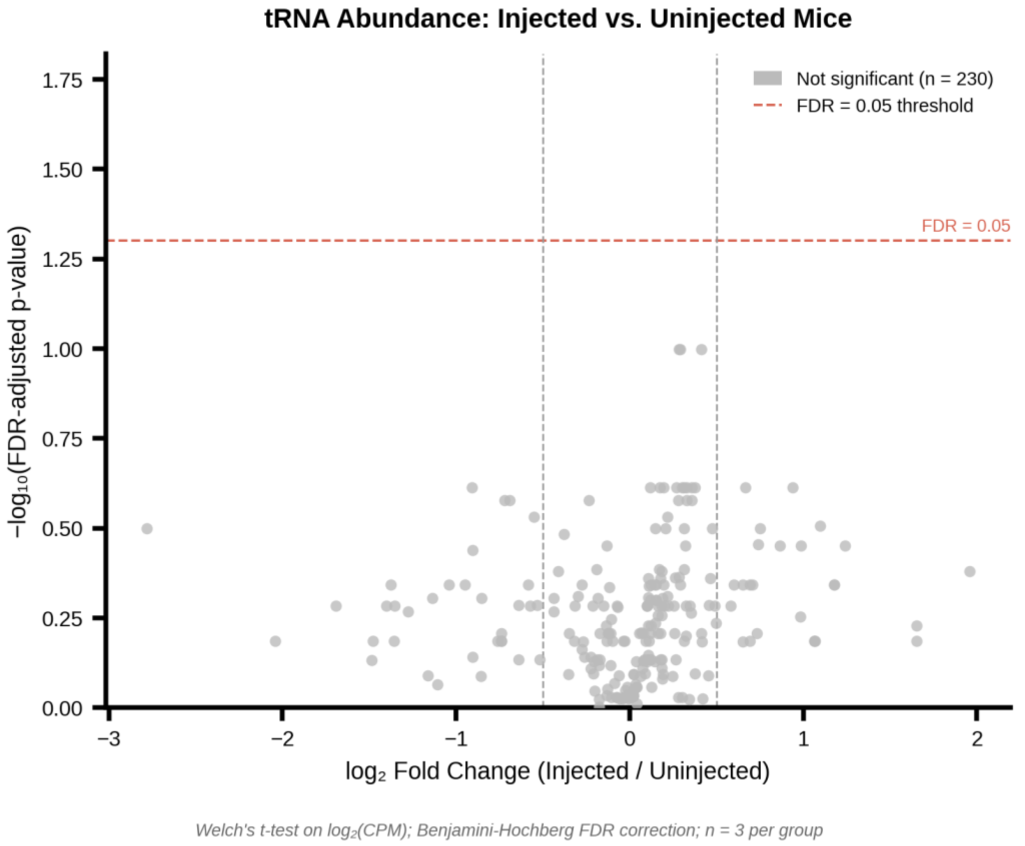

Figure S4. scAAV-delivered suppressor tRNA does not detectably perturb the endogenous tRNA pool. Volcano plot of endogenous tRNA abundance in brains from mice injected with scAAV-G(**I**) versus uninjected controls (n = 3 per group). Each point represents one endogenous tRNA species or isodecoder (n = 230). Counts were normalized to CPM, and differential abundance was tested by Welch's *t*-test on $\log_2$(CPM + 0.1), followed by Benjamini-Hochberg FDR correction. The x-axis shows $\log_2$ fold change; the y-axis shows $-\log_{10}$(FDR-adjusted *p*-value). Dashed vertical lines indicate ±0.5 $\log_2$ fold change, and the red dashed line marks FDR = 0.05. No endogenous tRNA was significantly altered after FDR correction.
